# Higher maternal BMI early in pregnancy is associated with overweight and obesity in young adult offspring in Thailand

**DOI:** 10.1186/s12889-021-10678-z

**Published:** 2021-04-14

**Authors:** Sakaewan Ounjaijean, Antika Wongthanee, Kanokwan Kulprachakarn, Amaraporn Rerkasem, Sakda Pruenglampoo, Ampica Mangklabruks, Kittipan Rerkasem, José G. B. Derraik

**Affiliations:** 1 NCD Center of Excellence, Research Institute for Health Sciences, Chiang Mai University, Chiang Mai, 50200 Thailand; 2 Department of Internal Medicine, Faculty of Medicine, Chiang Mai University, Chiang Mai, Thailand; 3 Department of Surgery, Faculty of Medicine, Chiang Mai University, Chiang Mai, Thailand; 4 Department of Women’s and Children’s Health, Uppsala University, Uppsala, Sweden; 5 Liggins Institute, University of Auckland, Auckland, New Zealand; 6 Children’s Hospital, Zhejiang University School of Medicine, Hangzhou, China

**Keywords:** Anthropometry, Body mass index, Developmental origins of health and disease, DOHaD, Metabolism, Mother, Programming, Weight

## Abstract

**Background:**

Rates of overweight and obesity among women of reproductive age have been steadily increasing worldwide and in Thailand. There is mounting evidence that maternal obesity during pregnancy is associated with an increased risk of obesity and other adverse health outcomes in the offspring, but such data are lacking for Thailand. We examined the associations between maternal body mass index (BMI) and anthropometry (particularly the likelihood of obesity) and cardiometabolic parameters in young adult offspring.

**Methods:**

This was a prospective follow-up study of a birth cohort in Chiang Mai (Thailand). Pregnant women carrying singletons were recruited at their first antenatal visit (< 24 weeks of gestation) and followed until delivery in 1989–1990. Participants were their young adult offspring followed up in 2010. Maternal BMI was recorded at the first antenatal visit. The offspring underwent clinical assessments, including anthropometry, lipid profile, insulin sensitivity (HOMA-IR), blood pressure, and carotid intima-media thickness. The primary outcome of interest was the likelihood of obesity in the offspring.

**Results:**

We assessed 628 young adults (54% were females) at 20.6 ± 0.5 years of age (range 19.1–22.1 years). The young adult offspring of mothers with overweight/obesity was 14.1 kg (95%CI 9.7, 18.5; *p* < 0.0001) and 9.4 kg (95% CI 6.1, 12.8; p < 0.0001) heavier than those born to mothers with underweight or normal weight, respectively, and had BMI 3.46 kg/m^2^ (95%CI 2.26, 4.67; *p* < 0.0001) and 5.27 kg/m^2^ (95%CI 3.67, 8.68; *p* < 0.0001) greater, respectively. For every 1-kg/m^2^ increase in maternal BMI, the adjusted odds ratio (aOR) of offspring obesity was 25% greater (95%CI 1.10, 1.42; *p* < 0.001). Thus, the aOR of obesity in offspring of mothers with overweight/obesity was 4.6 times greater (95%CI 1.86, 11.26; *p* < 0.001) and nearly 17-fold greater (95%CI 1.96, 146.4; *p* = 0.010) compared to young adults born to mothers with normal weight or underweight, respectively. There were no observed associations between maternal BMI status and offspring metabolism or blood pressure.

**Discussion:**

Maternal overweight/obesity early in pregnancy was associated with increased BMI and greater odds of obesity in their young adult offspring in Thailand. These findings highlight the public health importance of fostering healthier lifestyle choices among women of reproductive age.

## Background

Obesity is a growing public health issue worldwide. With the rise in obesity rates among women of reproductive age, there has been a consequent increase in the prevalence of women entering pregnancy with obesity [1, 2]. The prevalence of obesity [i.e., body mass index (BMI) ≥30.0 kg/m^2^] among pregnant women has been gradually increasing, from approximately 10% in the 1990s to 16–22% in the early 2000s [3, 4], and to as much as 30% in the present decade [1, 5, 6]. This rapid increase in the prevalence of pregnant women with overweight and obesity has been observed in both high- and middle-income countries [7], but the exact burden of overweight and obesity during pregnancy remains unclear.

Similar to what has been observed in other countries, rates of overweight and obesity in Thailand have been steadily increasing [8]. Data from the Thai National Health Examination Survey showed that rates of overweight (BMI 25.0–29.99 kg/m^2^) in young adult women (aged 18–24 years) increased from 8.6% in 1991 to 13.0% in 1997, and 18.1% in 2004; for obesity, the respective rates were 1.7, 4.9, and 5.7% [8]. However, there are limited data on rates of maternal overweight and obesity during pregnancy. A 2009 study in Bangkok reported that, at the first antenatal visit, 13 and 4% of 3715 pregnant women had overweight and obesity, respectively, [9]. Another study from a different hospital in Bangkok (2007–2010) reported very similar figures, with 14.2 and 3.7% of women entering pregnancy with overweight and obesity, respectively [10]. Lastly, data on 1192 pregnant women collected in 2006–2007 in Thailand’s four southernmost provinces (Songkhla, Pattani, Yala, and Narathiwat) again reported similar prevalence, with 15.5% having overweight and 3.4% obesity [11]. While there seems to be no published data looking at the trends in the prevalence of maternal obesity during pregnancy over the last decades in Thailand, these would most likely mirror the trends observed for young adult women in general.

Maternal obesity is associated with an increased risk of adverse health outcomes in pregnancy (e.g., miscarriage, gestational diabetes, and preeclampsia [12]), as well as both fetal and neonatal death [13]. Importantly, there is increasing evidence that maternal obesity is also associated with adverse long-term health outcomes in the offspring [14–16]. In particular, many studies have shown maternal obesity to be associated with an increased risk of obesity in the offspring in childhood, adolescence, and adulthood [17, 18]. However, the evidence for an association between maternal obesity and long-term obesity risk in the offspring has been mainly reported from Western countries. There is a paucity of data for Southeast Asia, particularly in adulthood, and the lack of research in low- and middle-income countries on the developmental origins of health and disease was recently highlighted by a systematic review on this subject [19].

In Thailand, several studies have reported on the associations between maternal obesity and adverse short-term outcomes during pregnancy and in the perinatal period [9, 20–22]. However, no studies in Thailand seem to have looked at associations between maternal obesity during pregnancy and potential long-term adverse health outcomes in the offspring. Therefore, we aimed to examine the associations between maternal BMI early in pregnancy and anthropometry and obesity risk in the adult offspring from a birth cohort in Thailand.

## Methods

### Study design

This was a prospective follow-up study of the offspring born to mothers from the Chiang Mai Low Birth Weight Study (1989–1990) in Thailand [23].

### Study participants

A total of 2184 pregnant women carrying singletons were recruited at their first antenatal visit (≤ 24 weeks of gestation) from two public hospitals in Chiang Mai, northern Thailand (Maharaj Nakorn Chiang Mai Hospital and The Maternal-Child Health Care Center). At the time, these were the only public hospitals providing antenatal care in Chiang Mai Province [23]. Participants were followed up through their routine clinical care until delivery.

In 2010, a follow-up study was carried out where our research team attempted to contact the mothers from the original study by phone, mail, and home visits, using available contact information from the Ministry of Interior and the Maharaj Nakorn Chiang Mai Hospital database [24]. Mothers and offspring who agreed to participate in the follow-up study underwent clinical assessments at the Research Institute for Health Sciences (RIHES) at Chiang Mai University.

### Assessments

Maternal weight and height were recorded at their first antenatal visit in the original study [23], at a median gestational age of 14 weeks [quartile 1 = 11 weeks, quartile 3 = 18 weeks]. Demographic characteristics were also obtained, including maternal and paternal education levels, and family income. Note that mother's gestational age in the original study was assessed from the reported last menstrual period and the fundal height; in cases of uncertainty, ultrasound measurements were also performed.

The young adults (offspring) had their height and weight measured while barefoot and wearing light clothing. BMI for both mother and offspring were derived, with BMI status categorised as underweight (BMI < 18.5 kg/m^2^), normal weight (BMI 18.5–24.99 kg/m^2^), overweight (BMI 25.0–29.99 kg/m^2^), or obesity (BMI ≥30.0 kg/m^2^), as defined by the World Health Organization [25].

Following an overnight fast, venous blood samples were collected from the young adult participants. Clinical parameters measured included lipid profile [total cholesterol, high-density lipoprotein cholesterol (HDL), and triglycerides], glucose, and insulin. The homeostatic model assessment of insulin resistance (HOMA-IR) was used as a surrogate marker of insulin sensitivity [26]. Laboratory assays were performed at RIHES.

Young adult participants had their blood pressure measured on the left arm at heart level using a sphygmomanometer, following a 5-min rest. Two measurements were taken, and the average was recorded. Carotid intima-media thickness (CIMT) was measured with a Philips iE33 and an L10–4 MHz linear array transducer. Measurements were made in the distal portion of the right common carotid artery while the participants were in the recumbent position. Note that in adults, CIMT is a known marker of cardiovascular health [27].

### Statistical analyses

Analyses were performed comparing health outcomes in the offspring of mothers stratified according to their BMI status early in pregnancy: Underweight, Normal weight, or Overweight/obesity (BMI ≥25.0 kg/m^2^). The primary outcome was the likelihood of obesity in young adult offspring. Secondary outcomes of interest included parameters on anthropometry (BMI and weight), glucose metabolism (HOMA-IR, fasting glucose, and fasting insulin), blood pressure (systolic and diastolic), lipid profile (total cholesterol, HDL, and triglycerides), and atherosclerosis marker (CIMT).

Data on demographic characteristics, maternal anthropometry, and birth parameters were compared using one-way ANOVA, non-parametric Kruskal-Wallis tests, or Fisher’s exact tests, as appropriate. The linear association between maternal BMI and offspring BMI was examined using Pearson’s correlation coefficients. Multivariable models were subsequently run, adjusting for several confounders known to affect anthropometry and health outcomes in young adulthood, namely gestational age [28–33], birth order [34–37], and sex [38]. The odds of obesity in the young adult offspring in association with maternal BMI were examined using generalised linear regression models (logistic regressions). Adjusted models were subsequently run, adjusting for the above-described confounders. 

Anthropometry and clinical parameters were then compared between young adult offspring born to mothers of different BMI statuses using general linear models. Multivariable models adjusted for the above-described confounders and the individual’s height where their weight or blood pressure [39] was the outcome, and maternal height where offspring height was the outcome.

Analyses were performed in SAS v9.4 (SAS Institute, Cary, USA) and SPSS v25 (IBM Corp, Armonk, NY, USA). All tests were two-tailed, with significance level maintained at *p* < 0.05, with no adjustments for multiple comparisons as per Rothman (1990) [40]. There were available data on the primary outcome for all included participants (i.e., completed anthropometric data), and missing data were not imputed.

## Results

### Study population

**Fig. 1:**
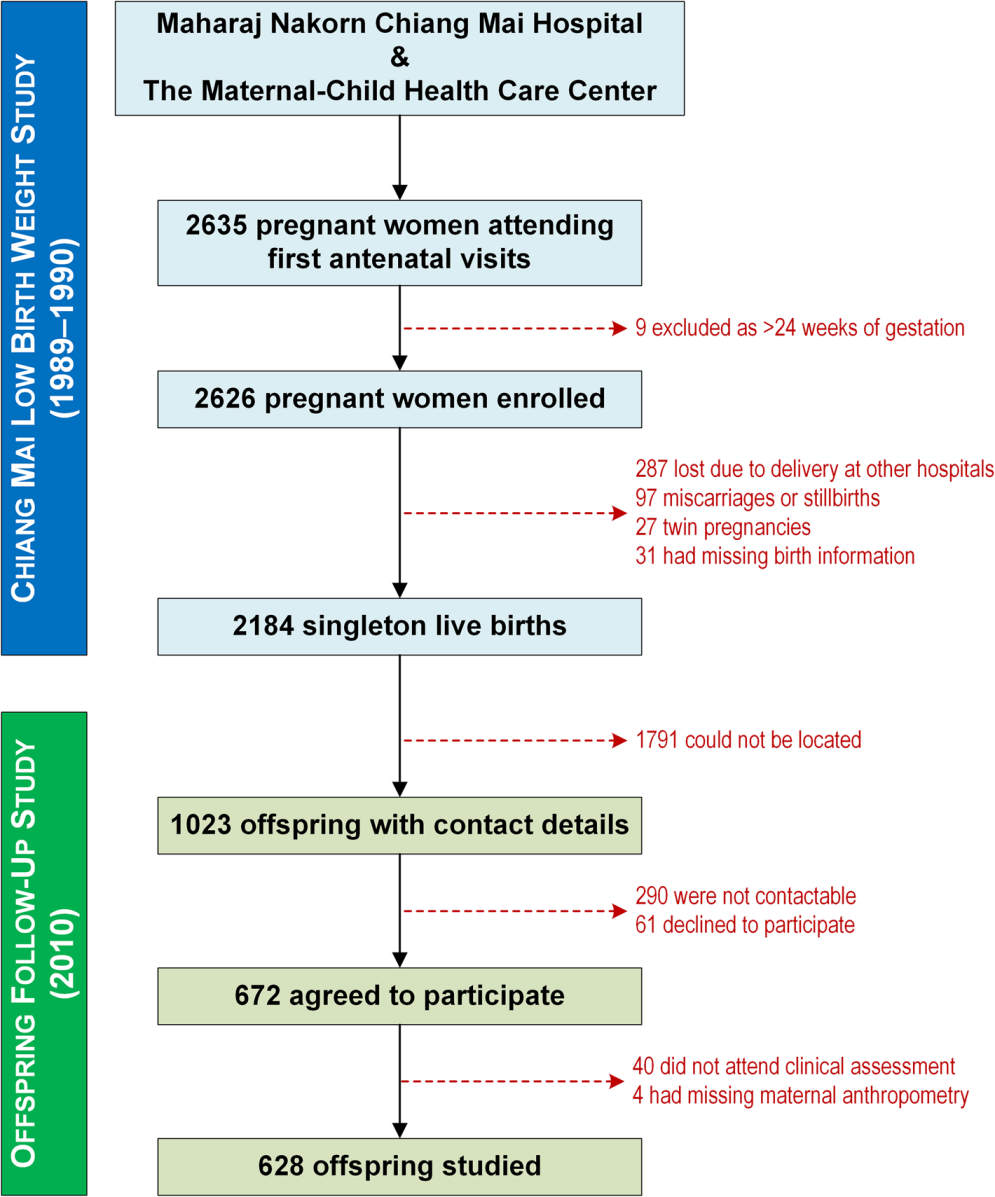
Flow diagram outlining participants’ recruitment into the Chiang Mai Low Birth Weight Study (1989–1990) and subsequently to the follow-up study on the offspring (2010)

From the 2184 mothers with liveborn infants in the original study, 672 agreed to participate; 632 young adults and their mothers attended the follow-up assessments and were enrolled in the study (Fig. 1). Four participants were excluded from this investigation due to incomplete maternal anthropometric data; thus, we studied 628 participants (338 females and 290 males) assessed at a mean age of 20.6 years (standard deviation = 0.5; range 19.1 to 22.1 years). Included and excluded participants had similar maternal or familial characteristics (data not shown) and similar mean birth weight (2.98 vs 3.01 kg, respectively; *p* = 0.09), but there were slight differences in birth length (48.8 vs 49.4 cm, respectively; *p* < 0.001) and gestational age (39.0 vs 38.8 weeks, respectively; *p* = 0.012).

The demographic and birth characteristics of our study population are shown in Table 1. Mothers who were underweight early in pregnancy were younger and better educated, and their partners were also better educated compared to mothers (and their partners) who were of normal weight or had overweight/obesity (Table 1). There was a progressive increase in infant birth weight according to maternal BMI status, with babies born to mothers with overweight/obesity being approximately 200 g and 360 g heavier than babies born to mothers who were of normal weight or underweight, respectively (Table 1). However, there were no observed differences in birth length or gestational age between the three groups of infants (Table 1).

**Table 1:** Demographic and birth characteristics of the study population according to maternal body mass index (BMI) status early in pregnancy

			Maternal BMI status			
	Characteristic		Underweight	Normal weight	Overweight/obesity	***P***-value
	n		62	513	53	
**Familial characteristics**	**Maternal BMI (kg/m**^**2**^**)**		17.84 [17.21, 18.16]	21.06 [19.95, 22.37]	26.43 [25.41, 27.54]	–
	**Maternal height (cm)**		153.2 ± 5.6	151.5 ± 4.9	151.2 ± 5.6	**0.034**
	**Maternal weight (kg)**		41.3 ± 3.0	48.8 ± 4.8	61.2 ± 5.9	**< 0.0001**
	**Maternal age at childbirth (years)**		24.1 ± 3.9	26.4 ± 4.6	28.4 ± 4.3	**< 0.0001**
	**Maternal education** ^**a**^	**Less than high school**	39 (81.3%)	400 (90.7%)	45 (97.8%)	**0.029**
		**High school or greater**	9 (18.8%)	41 (9.3%)	1 (2.2%)	
	**Paternal education** ^**b**^	**Less than high school**	31 (64.6%)	360 (81.6%)	42 (89.4%)	**0.007**
		**High school or greater**	17 (35.4%)	81 (18.4%)	5 (10.6%)	
	**Area of residence** ^**c**^	**Urban**	51 (83.6%)	400 (78.1%)	41 (80.4%)	0.64
		**Rural**	10 (16.4%)	112 (21.9%)	10 (19.6%)	
	**Family income (baht per month)** ^**d**^		3300 [2000, 5000]	2400 [1500, 4000]	2700 [1800, 4000]	0.08
**Offspring at birth**	**Sex**	**Female**	32 (51.6%)	276 (53.8%)	30 (56.6%)	0.86
		**Male**	30 (48.4%)	237 (46.2%)	23 (43.4%)	
	**Birth weight (kg)**		2.82 ± 0.56	2.98 ± 0.41	3.18 ± 0.38	**< 0.0001**
	**Birth length (cm)**		48.5 ± 3.1	48.7 ± 4.3	49.8 ± 2.2	0.19
	**Gestational age at delivery (weeks)**		38.9 ± 2.0	39.2 ± 1.7	39.4 ± 1.4	0.08
**Offspring at follow-up**	**Age (years)**		20.6 [20.3, 21.0]	20.6 [20.3, 20.9]	20.6 [20.3, 20.9]	0.24
	**Current smoking**		8 (12.9%)	57 (11.0%)	8 (15.1%)	0.55

Data are median [quartile 1, quartile 3]; mean ± standard deviation; or n (%), as appropriate

*P*-values for statistically significant differences (at *p* < 0.05) are shown in bold

^a^ *n* = 535 (85.2%)

^b^ *n* = 540 (85.4%)

^c^ Current area of residence of the offspring; *n* = 624 (99.4%)

^d^ Income recorded at the time of maternal recruitment to the original study in 1989–1990 (i.e. not adjusted for inflation)

### BMI status in mothers and offspring

The prevalence of obesity among mothers was minimal at 0.3%, while 8.1% were overweight (Table 2). In comparison, the prevalence of obesity in the offspring was 5.4% (18-fold greater) and of overweight 11.1% (Table 2), so that the prevalence of overweight/obesity was twice as high in the offspring than in mothers (16.5% vs 8.4%, respectively). Rates of obesity were the same in male and female offspring (5.5% vs 5.3%, respectively), but there was a greater proportion of males with overweight (14.5% vs 8.3%, respectively) (Table 2).

**Table 2:** Body mass index (BMI) status in two generations in Chiang Mai (Thailand): of the mothers early in pregnancy and young adult offspring at a mean age of 20.6 years

	Mothers	All offspring	Male offspring	Female offspring
**n**	628	628		
**Underweight**	62 (9.9%)	169 (26.9%)	57 (19.7%)	112 (33.1%)
**Normal weight**	513 (81.7%)	355 (56.5%)	175 (60.3%)	180 (53.3%)
**Overweight**	51 (8.1%)	70 (11.1%)	42 (14.5%)	28 (8.3%)
**Obesity**	2 (0.3%)	34 (5.4%)	16 (5.5%)	18 (5.3%)

Data are presented as n (%). Underweight, BMI < 18.5 kg/m^2^; normal weight, BMI 18.5–24.99 kg/m^2^; overweight, BMI 25.0–29.99 kg/m^2^; and obesity, BMI ≥30.0 kg/m^2^

### Offspring anthropometry

Increasing maternal BMI early in pregnancy was correlated with greater offspring weight (*r* = 0.23; *p* < 0.0001) and BMI (*r* = 0.25; *p* < 0.0001). Thus, after adjustment for confounders, the offspring of mothers with overweight/obesity were 9.4 kg heavier than the offspring born to mothers of normal weight (95% CI 6.1, 12.8; *p* < 0.0001) and 14.1 kg heavier than those born to underweight mothers (95% CI 9.7, 18.5; *p* < 0.0001) (Table 3). Similarly, the offspring of mothers with overweight/obesity had BMI that was 3.46 kg/m^2^ (95% CI 2.26, 4.67; *p* < 0.0001) and 5.27 kg/m^2^ (95% CI 3.67, 8.68; *p* < 0.0001) greater than young adults born to mothers of normal weight or who were underweight, respectively (Table 3). There were no associations between maternal BMI and offspring stature (Table 3).

**Table 3:** Anthropometric parameters in the offspring in association with maternal body mass index (BMI) status early in pregnancy, derived from both unadjusted and adjusted analyses

		Maternal BMI status		
		Underweight	Normal weight	Overweight/obesity
**Unadjusted**	**Height (cm)**	163.4 (161.3, 165.5)	164.0 (161.8, 166.2)	163.8 (163.1, 164.5)
	**Weight (kg)**	52.6 (46.6, 55.7)	57.2 (56.0, 58.4)^††^	66.7 (63.0, 70.3)^**** ††††^
	**BMI (kg/m**^**2**^**)**	19.4 (18.4, 20.5)	21.2 (20.8, 21.6)^††^	24.5 (23.5, 25.7)^**** ††††^
**Adjusted**	**Height (cm)**	163.0 (161.6, 164.4)	164.3 (163.8, 164.8)	165.1 (163.6, 166.5)
	**Weight (kg)**	52.3 (49.3, 55.3)	57.0 (56.0, 58.0)^††^	66.4 (63.3, 69.6)^**** ††††^
	**BMI (kg/m**^**2**^**)**	19.4 (18.3, 20.5)	21.2 (20.8, 21.5)^††^	24.6 (23.5, 25.8)^**** ††††^

Data are means and 95% confidence intervals

Underweight, BMI < 18.5 kg/m^2^; normal weight, 18.5–24.99 kg/m^2^; and overweight/obesity, ≥25.0 kg/m^2^

Adjusted models accounted for gestational age, birth order, sex, and age, as well as height for offspring weight, and maternal height for offspring height

^****^p < 0.0001 for comparisons to the offspring of mothers of normal weight; ^††^*p* < 0.01 and ^††††^*p* < 0.0001 for comparisons to the offspring of underweight mothers

**Table 4:** The unadjusted and adjusted odds ratios (OR) of obesity and overweight/obesity in the young adult offspring at a mean age of 20.6 years in association with maternal body mass index (BMI) status early in pregnancy

	OFFSPRING BMI STATUS			
	Overweight/obesity		Obesity	
**MATERNAL BMI STATUS**	**Unadjusted OR**	**Adjusted OR**	**Unadjusted OR**	**Adjusted OR**
**Overweight/obesity vs Underweight**	12.90 (3.57, 46.60)****	14.70 (3.95, 54.71)****	10.84 (1.31, 89.83)*	16.95 (1.96, 146.38)*
**Overweight/obesity vs Normal weight**	3.55 (1.95, 6.47)****	3.91 (2.10, 7.28)****	3.47 (1.48, 8.14)**	4.57 (1.86, 11.26)***
**Normal weight vs Underweight**	3.63 (1.11, 11.88)*	3.76 (1.14, 12.40)*	3.13 (0.42, 23.47)	3.70 (0.49, 28.06)

Data are the unadjusted and adjusted odds ratios with the respective 95% confidence intervals. Adjusted models accounted for gestational age, birth order, and sex

Underweight, BMI < 18.5 kg/m^2^; normal weight, BMI 18.5–24.99 kg/m^2^; and overweight/obesity, BMI ≥25.0 kg/m^2^

*p < 0.05, **p < 0.01, ***p < 0.001, and ****p < 0.0001 for pairwise comparisons

### Maternal BMI status vs offspring BMI status and the likelihood of obesity

**Fig. 2:**
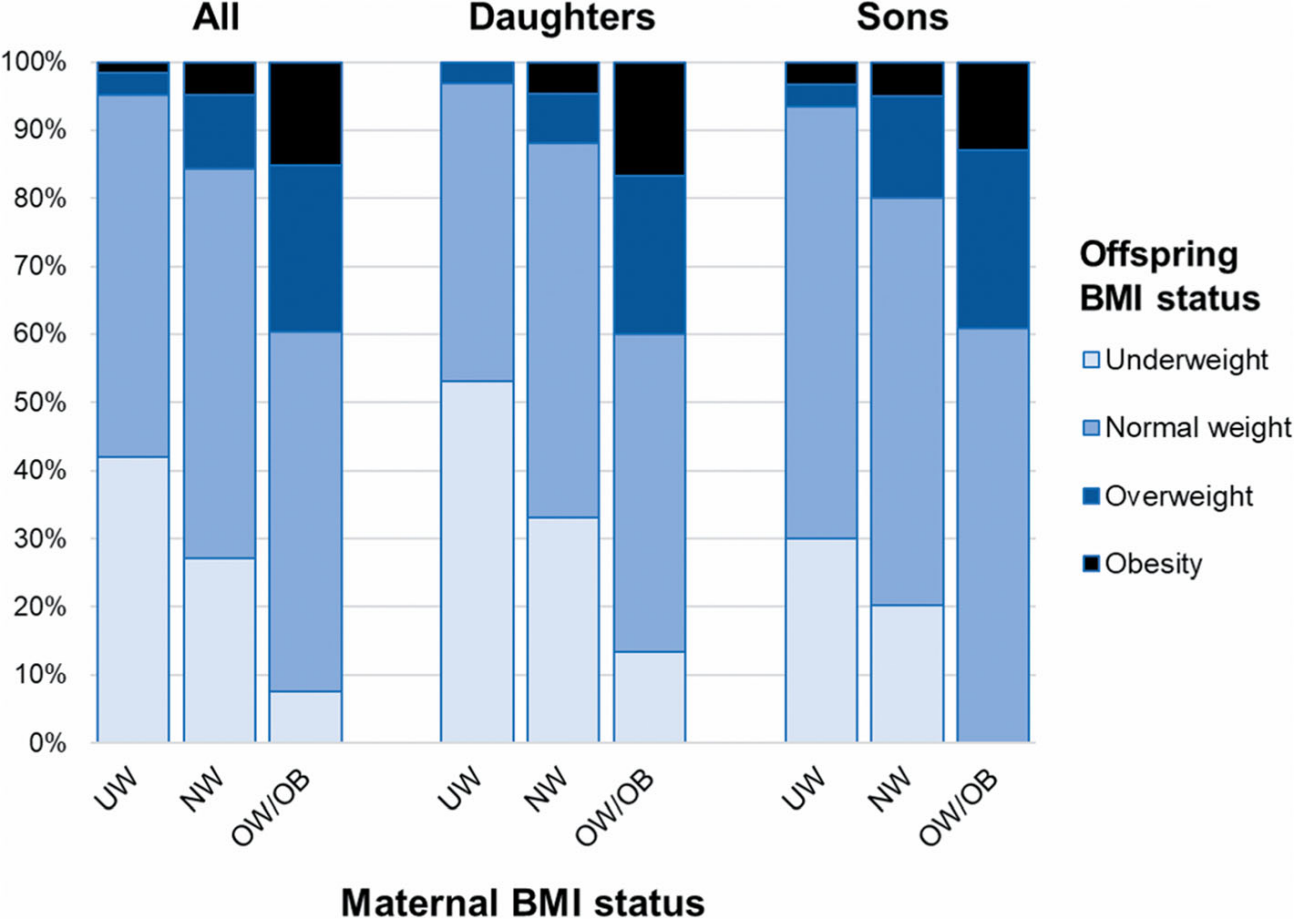
Body mass index (BMI) status in the young adult offspring (*n* = 628) at a mean age of 20.6 years in Chiang Mai (Thailand), according to maternal BMI status early in pregnancy. Underweight, BMI < 18.5 kg/m^2^; normal weight, BMI 18.5–24.99 kg/m^2^; overweight, BMI 25.0–29.99 kg/m^2^; obesity, BMI ≥30.0 kg/m^2^; and overweight/obesity, BMI ≥25.0 kg/m^2^. NW: mothers with normal weight; OW/OB: mothers with overweight/obesity; UW, mothers with underweight

The associations between mothers’ BMI status early in pregnancy and the BMI status of their young adult offspring are presented in Fig. 2. Rates of overweight and rates of obesity in the offspring were progressively higher with increasing maternal BMI status, with the same pattern observed in males and females (Fig. 2).

For every 1 kg/m^2^ increase in maternal BMI early in pregnancy, the adjusted odds ratio (aOR) of obesity in the offspring was 25% greater [aOR 1.25 (95% CI 1.10, 1.42); *p* < 0.001]. As a result, the aOR of obesity in the offspring of mothers with overweight/obesity was 4.6 times greater (95% CI 1.86, 11.26; *p* < 0.001) and 17-fold greater (95% CI 1.96, 146.38; *p* = 0.010) than in young adults born to mothers who were of normal weight or underweight, respectively (Table 4).

### Cardiometabolic outcomes

There were no observed associations between maternal BMI status early in pregnancy and offspring metabolism or blood pressure (Table 5). The exception was an isolated (and likely spurious) finding of total cholesterol/HDL that was 9% lower in the offspring of mothers with normal weight compared to those born to mothers with overweight/obesity (*p* = 0.024; Table 5).

**Table 5:** Cardiometabolic outcomes in the young adult offspring at a mean age of 20.6 years in association with maternal body mass index (BMI) status early in pregnancy, derived from both unadjusted and adjusted analyses

		UNADJUSTED			ADJUSTED		
		Underweight	Normal weight	Overweight/obesity	Underweight	Normal weight	Overweight/obesity
**Glucose metabolism**	**n (%)**	62 (100%)	497 (97%)	51 (96%)	62 (100%)	497 (97%)	51 (96%)
	**Fasting glucose (mg/dL)**	83 (81, 85)	83 (82, 83)	83 (81, 85)	83 (81, 85)	83 (82, 84)	83 (81, 86)
	**Fasting insulin (mIU/L)**	7.34 (6.29, 8.79)	7.24 (6.83, 7.68)	8.41 (6.99, 10.12)	7.21 (6.04, 8.61)	7.15 (6.74, 7.60)	8.72 (7.21, 10.55)
	**HOMA-IR**	1.53 (1.28, 1.82)	1.48 (1.40, 1.58)	1.72 (1.42, 2.08)	1.49 (1.24, 1.79)	1.47 (1.38, 1.56)	1.79 (1.47, 2.18)
**Atherosclerosis marker**	**n (%)**	61 (98%)	488 (95%)	51 (96%)	61 (98%)	488 (95%)	51 (96%)
	**CIMT (mm)**	0.443 (0.435. 0.451)	0.439 (0.436, 0.442)	0.436 (0.428, 0.445)	0.445 (0.436, 0.453)	0.440 (0.437, 0.443)	0.435 (0.426, 0.444)
**Blood pressure**	**n (%)**	62 (100%)	504 (98%)	52 (98%)	62 (100%)	504 (98%)	52 (98%)
	**Systolic (mmHg)**	114 (111, 117)	115 (113, 116)	116 (112, 119)	114 (111, 117)	115 (114, 116)	116 (113, 119)
	**Diastolic (mmHg)**	74 (71, 77)	74 (73, 75)	74 (71, 77)	74 (72, 77)	74 (73, 75)	74 (71, 77)
**Lipid profile**	**n (%)**	62 (100%)	497 (97%)	52 (98%)	62 (100%)	497 (97%)	52 (98%)
	**Total cholesterol (mg/dL)**	168 (159, 176)	168 (165, 171)	174 (165, 184)	167 (157, 176)	167 (164, 170)	173 (163, 183)
	**HDL (mg/dL)**	55 (52, 59)	57 (55, 58)	54 (50, 58)	55 (51, 59)	57 (55, 58)	53 (49, 57)
	**Triglycerides (mg/dL)**	82 (73, 93)	75 (72, 79)	75 (65, 85)	82 (72, 93)	76 (72, 79)	76 (66, 88)
	**Total cholesterol/HDL**	3.13 (2.91, 3.35)	3.11 (3.03, 3.19)*	3.36 (3.12, 3.59)	3.13 (2.90, 3.36)	3.11 (3.03, 3.18)*	3.41 (3.16, 3.64)

CIMT, carotid intima-media thickness; HDL, high-density lipoprotein cholesterol; HOMA-IR, homeostatic model assessment of insulin resistance

Underweight, BMI < 18.5 kg/m^2^; normal weight, BMI 18.5–24.99 kg/m^2^; and overweight/obesity, BMI ≥25.0 kg/m^2^

Data on n (%) are the number and proportion of available samples for a given parameter per group; all other data are means and the respective 95% confidence intervals, with adjusted models accounting for gestational age, birth order, and sex, as well as participant’s height for blood pressure

*p < 0.05 for a pairwise comparison to the offspring of mothers with overweight/obesity

## Discussion

This study shows that maternal overweight/obesity early in pregnancy was associated with a marked increase in the likelihood of obesity in the young adult offspring in Thailand. These findings in Thai people corroborate the body of evidence mostly from Western countries reported in childhood, late adolescence, and adulthood [17, 18], which show that maternal obesity begets obesity in the offspring [41].

Of note, we observed no associations between maternal BMI status early in pregnancy and cardiometabolic outcomes in the young adult offspring. As reviewed by Drake & Reynolds, studies have reported associations between maternal obesity during pregnancy and adverse cardiometabolic health in their offspring, including dysregulation of glucose/insulin homoeostasis and vascular dysfunction [42]. A large British study on 37,709 people also showed that maternal obesity during pregnancy was associated with increased risk of hospitalisation and all-cause mortality in the offspring aged 34–61 years [43]. It is possible that our cohort was still too young, and therefore yet to develop overt cardiometabolic dysfunction. Therefore, it would be of interest to follow-up our participants in the long-term to ascertain whether their young age was a factor, or whether there may be inherent differences between our study population in Thailand and other groups studied overseas that mainly consisted of Caucasians.

There are still uncertainties regarding the potential mechanisms underpinning the effects of maternal obesity on offspring obesity risk and long-term health. Animal models have attempted to describe these mechanisms [44, 45], and this association between maternal and offspring obesity risk has been explained, at least in part, by shared genetic traits that influence body weight or weight gain [46, 47]. It has been suggested that a combination of changes in fetal nutrient supply and genetic and epigenetic mechanisms may be at play [48].

In women with overweight or obesity, the effects of gestational weight gain on obesity risk in the offspring are likely associated with mechanisms in utero, in contrast to mothers of normal weight, where such effects likely result from shared familial characteristics (i.e., genes and the early environmental) [49]. It is also possible that the expected changes in maternal metabolism during pregnancy are exacerbated in women with obesity, leading to increased inflammation and higher blood lipids levels, which, in turn, alter the development of the embryo and fetus in utero [48]. Further, Catalano proposed that the increased maternal insulin resistance early in pregnancy due to maternal obesity may be associated with altered placental function and increased fetoplacental availability of nutrients later in gestation, not only of glucose but also of free fatty acids and amino acids [41].

It should be noted that beyond maternal obesity, there are other potential reasons for the observed increase in the overall rates of overweight and obesity in the offspring, including among those born to underweight mothers. For example, this increase might be related to lifestyle changes in the offspring, most notably regarding dietary patterns due to Thailand’s economic and social transitions since the mid-1980s. Thai people’s dietary patterns have shifted from their traditional rice-based diet, low in fat, and with a high intake of vegetables, to a westernised diet based on beef and pork, high in fats and simple sugars [50]. Besides, with increasing urbanisation, Thai people have progressively lessened their leisure and work-related physical activity levels, spending more time in sedentary activities, such as watching television, ‘surfing’ the internet, and playing video games [51]. Not surprisingly, a nationwide study on 87,134 students (median age 29 years) from an open university in Thailand reported increased odds of obesity in association with a range of lifestyle factors; these included lower levels of self-reported physical activity, increased sedentary behaviours (e.g., watching television or spending time on computers), and consumption of unhealthy foods (e.g., fried foods, Western-style fast food, or soft drinks) [52].

Our study’s main limitation was the lack of data on lifestyle parameters in the offspring, particularly physical activity levels and dietary intake. Nonetheless, key demographic parameters associated with obesity risk (i.e., parental education and family income) were relatively similar in the groups stratified according to maternal BMI. We also did not have data on paternal anthropometry; while maternal BMI is a stronger determinant of offspring obesity than paternal BMI [53], there is evidence that elevated BMI in both mothers and fathers has a compounding effect on offspring obesity risk [53, 54]. Lastly, mothers in our study had their BMI status derived based on measurements in the first trimester of pregnancy. However, obtaining pre-pregnancy data is difficult, as most pregnancies are unplanned, and most women do not seek pre-conceptional care [55–57]. Not surprisingly, most studies comparing BMI at the first antenatal visit and pre-pregnancy BMI have relied mainly on self-reported pre-pregnancy weight and height [55–58], observing only minor differences between the two BMI measures. Few studies have compared actual pre-pregnancy measurements to those at the first antenatal visit. While two investigations have identified discrepancies in BMI classification between pre-pregnancy and the first trimester of pregnancy of 5–9% [59] and 10% [60], a study on 1000 women reported that maternal weight and body composition were essentially unchanged throughout the first trimester of pregnancy [61]. Thus, although it is likely that the BMI status of a few women in our study would have been misclassified in comparison to their pre-pregnancy status, we contend that BMI status classification based on measurements in the first trimester of pregnancy are largely reliable. Among the strengths of our study, apart from its prospective design, this investigation is of particular relevance as ours appears to be the first study to examine long-term associations between maternal BMI during pregnancy and long-term health in the offspring in Thailand. Further, our study participants underwent a range of cardiometabolic assessments that provided a relatively comprehensive assessment of their health beyond anthropometric measurements.

## Conclusions

Despite the growing evidence that maternal obesity adversely affects the offspring’s long-term health, the rates of women entering pregnancy with obesity continue to increase [46]. Our study adds further evidence on this problem, showing that it also affects non-Western countries such as Thailand. Therefore, greater recognition of the impacts of maternal obesity on the health of future generations is required to inform public health policy and intervention, particularly to foster healthier lifestyle choices among women of childbearing age (i.e., before conception).

## Data Availability

The study data cannot be made available in a public repository due to the conditions of the ethics approval. However, the anonymised data on which this manuscript was based could be made available to other investigators upon bona fide request, and following all the necessary approvals (including ethics) of the detailed study proposal and statistical analyses plan. Any queries should be directed to the corresponding authors.

## Abbreviations

aOR: Adjusted odds ratio
BMI: Body mass index
CI: Confidence interval
CIMT: Carotid intima-media thickness
HOMA-IR: Homeostatic model assessment of insulin resistance
RIHES: Research Institute for Health Sciences

## References

[1] Huda SS, Brodie LE, Sattar N (2010). Obesity in pregnancy: prevalence and metabolic consequences. Semin Fetal Neonatal Med.

[2] Ng M, Fleming T, Robinson M, Thomson B, Graetz N, Margono C, et al. Global, regional, and national prevalence of overweight and obesity in children and adults during 1980-2013: a systematic analysis for the global burden of disease study 2013. Lancet. 2014;384(9945):766–81. 10.1016/S0140-6736(14)60460-8. 10.1016/S0140-6736(14)60460-8 PMC4624264 24880830

[3] Kim SY, Dietz PM, England L, Morrow B, Callaghan WM (2007). Trends in pre-pregnancy obesity in nine states, 1993-2003. Obesity.

[4] Heslehurst N, Ells LJ, Simpson H, Batterham A, Wilkinson J, Summerbell CD (2007). Trends in maternal obesity incidence rates, demographic predictors, and health inequalities in 36,821 women over a 15-year period. BJOG.

[5] Flegal KM, Carroll MD, Kit BK, Ogden CL (2012). Prevalence of obesity and trends in the distribution of body mass index among US adults, 1999-2010. JAMA.

[6] Devlieger R, Benhalima K, Damm P, Van Assche A, Mathieu C, Mahmood T, et al. Maternal obesity in Europe: where do we stand and how to move forward?: a scientific paper commissioned by the European Board and College of Obstetrics and Gynaecology (EBCOG). Eur J Obstet Gynecol Reprod Biol. 2016;201:203–8. 10.1016/j.ejogrb.2016.04.005. 10.1016/j.ejogrb.2016.04.005 27160501

[7] Chen C, Xu X, Yan Y (2018). Estimated global overweight and obesity burden in pregnant women based on panel data model. PLoS One.

[8] Aekplakorn W, Mo-suwan L. Prevalence of obesity in Thailand. Obes Rev. 2009;10(6):589–92. 10.1111/j.1467-789X.2009.00626.x. 10.1111/j.1467-789X.2009.00626.x 19656310

[9] Saereeporncharenkul K (2011). Correlation of BMI to pregnancy outcomes in Thai women delivered in Rajavithi hospital. J Med Assoc Thail.

[10] Titapant V (2013). Is the U.S. Institute of Medicine recommendation for gestational weight gain suitable for Thai singleton pregnant women?. J Med Assoc Thail.

[11] Liabsuetrakul T (2011). Is international or Asian criteria-based body mass index associated with maternal anaemia, low birthweight, and preterm births among Thai population? An observational study. J Health Popul Nutr.

[12] Mamun AA, Callaway LK, O'Callaghan MJ, Williams GM, Najman JM, Alati R, Clavarino A, Lawlor DA (2011). Associations of maternal pre-pregnancy obesity and excess pregnancy weight gains with adverse pregnancy outcomes and length of hospital stay. BMC Pregnancy Childbirth.

[13] Aune D, Saugstad OD, Henriksen T, Tonstad S (2014). Maternal body mass index and the risk of fetal death, stillbirth, and infant death: a systematic review and meta-analysis. JAMA.

[14] Catalano PM, Shankar K (2017). Obesity and pregnancy: mechanisms of short term and long term adverse consequences for mother and child. BMJ.

[15] Valsamakis G, Kyriazi EL, Mouslech Z, Siristatidis C, Mastorakos G (2015). Effect of maternal obesity on pregnancy outcomes and long-term metabolic consequences. Hormones.

[16] Ford SP, Tuersunjiang N (2013). Maternal obesity: how big an impact does it have on offspring prenatally and during postnatal life?. Expert Rev Endocrinol Metab.

[17] Derraik JGB, Ahlsson F, Diderholm B, Lundgren M (2015). Obesity rates in two generations of Swedish women entering pregnancy, and associated obesity risk among adult daughters. Sci Rep.

[18] Tequeanes AL, Gigante DP, Assunção MC, Chica DA, Horta BL. Maternal anthropometry is associated with the body mass index and waist:height ratio of offspring at 23 years of age. J Nutr. 2009;139(4):750–4. 10.3945/jn.108.100669. 10.3945/jn.108.100669 19211832

[19] Tu'akoi S, Vickers MH, Bay JL (2020). DOHaD in low- and middle-income countries: a systematic review exploring gaps in DOHaD population studies. J Dev Orig Health Dis.

[20] Fang R, Dawson A, Lohsoonthorn V, Williams MA (2009). Risk factors of early and late onset preeclampsia among Thai women. Asian Biomed (Res Rev News).

[21] Pongcharoen T, Gowachirapant S, Wecharak P, Sangket N, Winichagoon P (2016). Pre-pregnancy body mass index and gestational weight gain in Thai pregnant women as risks for low birth weight and macrosomia. Asia Pac J Clin Nutr.

[22] Kongubol A, Phupong V (2011). Prepregnancy obesity and the risk of gestational diabetes mellitus. BMC Pregnancy Childbirth.

[23] Chiang Mai Low Birth Weight Study Group (2012). The risk factors of low birth weight infants in the northern part of Thailand. J Med Assoc Thail.

[24] Rerkasem K, Wongthanee A, Rerkasem A, Chiowanich P, Sritara P, Pruenglampoo S, Mangklabruks A (2012). Intrauterine nutrition and carotid intimal media thickness in young Thai adults. Asia Pac J Clin Nutr.

[25] WHO (2000). Obesity: preventing and managing the global epidemic. Report of a WHO consultation. World Health Organ Tech Rep Ser.

[26] Wallace TM, Levy JC, Matthews DR (2004). Use and abuse of HOMA modeling. Diabetes Care.

[27] Bots ML, Hoes AW, Koudstaal PJ, Hofman A, Grobbee DE (1997). Common carotid intima-media thickness and risk of stroke and myocardial infarction: the Rotterdam study. Circulation.

[28] Mathai S, Cutfield WS, Derraik JGB, Dalziel SR, Harding JE, Robinson E, Biggs J, Jefferies C, Hofman PL (2012). Insulin sensitivity and β-cell function in adults born preterm and their children. Diabetes.

[29] Mathai S, Derraik JGB, Cutfield WS, Dalziel SR, Harding JE, Biggs JB, Jefferies C, Hofman PL (2015). Blood pressure abnormalities in adults born moderately preterm and their children. Int J Cardiol.

[30] Skudder-Hill L, Ahlsson F, Lundgren M, Cutfield WS, Derraik JGB (2019). Preterm birth is associated with increased blood pressure in young adult women. J Am Heart Assoc.

[31] Sipola-Leppänen M, Vääräsmäki M, Tikanmäki M, Matinolli H-M, Miettola S, Hovi P, et al. Cardiometabolic risk factors in young adults who were born preterm. Am J Epidemiol. 2015;181(11):861–73. 10.1093/aje/kwu443. 10.1093/aje/kwu443 PMC4445394 25947956

[32] Ayyavoo A, Derraik JGB, Hofman PL, Mathai S, Biggs J, Stone P, Sadler L, Cutfield WS (2013). Pre-pubertal children born post-term have reduced insulin sensitivity and other markers of the metabolic syndrome. PLoS One.

[33] Derraik JGB, Lundgren M, Cutfield WS, Ahlsson F (2016). Body mass index, overweight, and obesity in Swedish women born post-term. Paediatr Perinat Epidemiol.

[34] Jelenkovic A, Silventoinen K, Tynelius P, Myrskyla M, Rasmussen F (2013). Association of birth order with cardiovascular disease risk factors in young adulthood: a study of one million Swedish men. PLoS One.

[35] Aurpibul L (2021). Butler É M, Wongthanee a, Rerkasem a, Pruenglampoo S, Mangklabruks a, Rerkasem K, Derraik JGB: birth order is associated with an increased risk of obesity in young adults in Thailand. J Epidemiol Community Health.

[36] Siervo M, Stephan B, Colantuoni A, Wells J (2011). First-borns have a higher metabolic rate and carry a higher metabolic risk in young women attending a weight loss clinic. Eat Weight Disord.

[37] Derraik JGB, Ahlsson F, Lundgren M, Jonsson B, Cutfield WS. First-borns have greater BMI and are more likely to be overweight or obese: a study of sibling pairs among 26,812 Swedish women. J Epidemiol Community Health. 2016;70(1):78–81. 10.1136/jech-2014-205368. 10.1136/jech-2014-205368 26311896

[38] Gabory A, Roseboom TJ, Moore T, Moore LG, Junien C (2013). Placental contribution to the origins of sexual dimorphism in health and diseases: sex chromosomes and epigenetics. Biol Sex Diff.

[39] Bourgeois B, Watts K, Thomas DM, Carmichael O, Hu FB, Heo M, Hall JE, Heymsfield SB (2017). Associations between height and blood pressure in the United States population. Medicine.

[40] Rothman KJ (1990). No adjustments are needed for multiple comparisons. Epidemiology.

[41] Catalano PM (2003). Obesity and pregnancy – the propagation of a viscous cycle?. J Clin Endocrinol Metab.

[42] Drake AJ, Reynolds RM (2010). Impact of maternal obesity on offspring obesity and cardiometabolic disease risk. Reproduction.

[43] Reynolds RM, Allan KM, Raja EA, Bhattacharya S, McNeill G, Hannaford PC, et al. Maternal obesity during pregnancy and premature mortality from cardiovascular event in adult offspring: follow-up of 1 323 275 person years. BMJ. 2013;347:f4539. 10.1136/bmj.f4539. 10.1136/bmj.f4539 PMC3805484 23943697

[44] Eaton SA, Aiken AJ, Young PE, Ho JWK, Cropley JE, Suter CM (2018). Maternal obesity heritably perturbs offspring metabolism for three generations without serial programming. Int J Obes.

[45] Huypens P, Sass S, Wu M, Dyckhoff D, Tschop M, Theis F, et al. Epigenetic germline inheritance of diet-induced obesity and insulin resistance. Nat Genet. 2016;48(5):497–9. 10.1038/ng.3527. 10.1038/ng.3527 26974008

[46] Thaker VV (2017). Genetic and epigenetic causes of obesity. Adolesc Med State Art Rev.

[47] Chamorro-Garcia R, Blumberg B (2014). Transgenerational effects of obesogens and the obesity epidemic. Curr Opin Pharmacol.

[48] Heerwagen MJ, Miller MR, Barbour LA, Friedman JE (2010). Maternal obesity and fetal metabolic programming: a fertile epigenetic soil. Am J Physiol Regul Integr Comp Physiol.

[49] Lawlor DA, Lichtenstein P, Fraser A, Långström N (2011). Does maternal weight gain in pregnancy have long-term effects on offspring adiposity? A sibling study in a prospective cohort of 146,894 men from 136,050 families. Am J Clin Nutr.

[50] Aekplakorn W, Satheannoppakao W, Putwatana P, Taneepanichskul S, Kessomboon P, Chongsuvivatwong V, Chariyalertsak S (2015). Dietary pattern and metabolic syndrome in thai adults. J Nutr Metab.

[51] Jitnarin N, Kosulwat V, Rojroongwasinkul N, Boonpraderm A, Haddock CK, Poston WS (2011). Prevalence of overweight and obesity in Thai population: results of the National Thai Food Consumption Survey. Eat Weight Disord.

[52] Banwell C, Lim L, Seubsman SA, Bain C, Dixon J, Sleigh A (2009). Body mass index and health-related behaviours in a national cohort of 87,134 Thai open university students. J Epidemiol Community Health.

[53] Zalbahar N, Najman J, McIntrye HD, Mamun A (2016). Parental pre-pregnancy BMI influences on offspring BMI and waist circumference at 21 years. Aust N Z J Public Health.

[54] Næss M, Holmen TL, Langaas M, Bjørngaard JH, Kvaløy K (2016). Intergenerational transmission of overweight and obesity from parents to their adolescent offspring – the HUNT study. PLoS One.

[55] Natamba BK, Sanchez SE, Gelaye B, Williams MA (2016). Concordance between self-reported pre-pregnancy body mass index (BMI) and BMI measured at the first prenatal study contact. BMC Pregnancy Childbirth.

[56] Finer LB, Zolna MR (2014). Shifts in intended and unintended pregnancies in the United States, 2001-2008. Am J Public Health.

[57] Shin D, Chung H, Weatherspoon L, Song WO (2014). Validity of prepregnancy weight status estimated from self-reported height and weight. Matern Child Health J.

[58] Holland E, Moore Simas TA, Doyle Curiale DK, Liao X, Waring ME (2013). Self-reported pre-pregnancy weight versus weight measured at first prenatal visit: effects on categorisation of pre-pregnancy body mass index. Matern Child Health J.

[59] Krukowski RA, West DS, DiCarlo M, Shankar K, Cleves MA, Saylors ME, Andres A (2016). Are early first trimester weights valid proxies for preconception weight?. BMC Pregnancy Childbirth.

[60] Gilmore LA, Redman LM (2015). Weight gain in pregnancy and application of the 2009 IOM guidelines: toward a uniform approach. Obesity.

[61] Fattah C, Farah N, Barry SC, O'Connor N, Stuart B, Turner MJ (2010). Maternal weight and body composition in the first trimester of pregnancy. Acta Obstet Gynecol Scand.

[62] Rerkasem K, Kulprachakarn K, Wongthanee A, Rerkasem A, Chiowanich P, Pruenglampoo S, Mangklabruks A, Sritara P, Derraik JGB (2019). Maternal overweight/obesity is associated with markedly greater odds of obesity in the offspring at 20 years of age in Thailand. J Dev Orig Health Dis.

[63] World Medical Association (2013). World medical association declaration of Helsinki: ethical principles for medical research involving human subjects. JAMA.

